# Collagen mRNA levels changes during colorectal cancer carcinogenesis

**DOI:** 10.1186/1471-2407-9-136

**Published:** 2009-05-07

**Authors:** Hanne Skovbjerg, Dorit Anthonsen, Inger MB Lothe, Kjell M Tveit, Elin H Kure, Lotte K Vogel

**Affiliations:** 1 Medical Department, Amager Hospital, Copenhagen S, Denmark; 2 Department of Cellular and Molecular Medicine, Faculty of Health Science, University of Copenhagen, Copenhagen, Denmark; 3 Department of Cellular and Molecular Medicine, Faculty of Health Science, University of Copenhagen, Copenhagen, Denmark; 4 The Cancer Centre, Ulleval University Hospital, Oslo, Norway; 5 Department of Pathology, Ulleval University Hospital, Oslo, Norway; 6 Department of Environmental and Health Studies, Telemark University College, Bø, Norway

## Abstract

**Background:**

Invasive growth of epithelial cancers is a complex multi-step process which involves dissolution of the basement membrane. Type IV collagen is a major component in most basement membranes. Type VII collagen is related to anchoring fibrils and is found primarily in the basement membrane zone of stratified epithelia. Immunohistochemical studies have previously reported changes in steady-state levels of different α(IV) chains in several epithelial cancer types. In the present study we aimed to quantitatively determine the mRNA levels of *type IV collagen (α1/α4/α6) *and *type VII collagen (α1) *during colorectal cancer carcinogenesis.

**Methods:**

Using quantitative RT-PCR, we have determined the mRNA levels for *α1(IV), α4(IV), α6(IV), and α1(VII) *in colorectal cancer tissue (n = 33), adenomas (n = 29) and in normal tissue from the same individuals. In addition, corresponding tissue was examined from healthy volunteers (n = 20). mRNA levels were normalized to *β-actin*. Immunohistochemical analysis of the distributions of type IV and type VII collagens were performed on normal and affected tissues from colorectal cancer patients.

**Results:**

The *α1(IV) *and *α1(VII) *mRNA levels were statistically significantly higher in colorectal cancer tissue (p < 0.001) as compared to corresponding tissue from healthy controls. This is an early event as tissue from adenomas also displayed a higher level. There were small changes in the levels of *α4(IV)*. The level of *α6(IV) *was 5-fold lower in colorectal cancer tissue as compared to healthy individuals (p < 0.01). The localisation of type IV and type VII collagen was visualized by immunohistochemical staining.

**Conclusion:**

Our results suggest that the down-regulation of *α6(IV*) mRNA coincides with the acquisition of invasive growth properties, whereas *α1(IV) *and *α1(VII) *mRNAs were up-regulated already in dysplastic tissue. There are no differences in collagen expression between tissues from healthy individuals and normal tissues from affected individuals.

## Background

The basement membrane (BM) acts as a barrier separating the epithelium from the underlying stroma. BMs are composed of a number of collagens and non-collageneous proteins. Type IV collagen is a major component and is present ubiquitously in all BMs [1,2]. Type IV collagen is composed of six genetically distinct chains (α1(IV) to α6(IV)) assembled in triple helical domains. Three molecular forms of type IV collagen, composed of α1(IV)_2_/α2(IV), α3(IV)/α4(IV)/α5(IV) and α5(IV)_2_/α6(IV), have been characterized. The α1(IV) and α2(IV) chains are widely distributed and found in all BMs of the whole body whereas the α3(IV) – α6(IV) chains are distributed in the BMs in a tissue-specific manner [3-6]. All six type IV collagen α-chains have been identified in the human colon by immunofluorescence staining using chain-specific monoclonal antibodies [7]. Invasive growth is a hallmark of malignancy in cancers and several studies suggest that the loss of tissue specific α(IV) chains in the epithelial BM may be related to biologically significant events in the invasive stage of cancer in different tissues [8-17].

Type VII collagen is related to BM anchoring fibrils and is composed of three identical α chains in a triple helical domain [18]. It is in addition to type IV collagen an important component of collagen under stratified squamous epithelium. A few studies have demonstrated the presence of type VII collagen in normal colon as a linear staining of the subepithelial BM [19,20] or in dysplastic epithelium [17]. It has been suggested that type VII collagen is involved in the process of early invasion [17].

In the previous studies, the distributions of type IV and type VII collagen in normal and colorectal cancer tissue have mainly been studied by use of immunohistochemical methods – a technique that allows a semi-quantitative measure of the protein steady state level.

In the present study we have for the first time investigated the mRNA levels of *type IV collagen (α1(IV), α4(IV), α6(IV))*, and *type VII collagen *in affected and normal tissue from individuals with colorectal adenomas and carcinomas as well as in normal tissue from healthy individuals. Our aim was to investigate which changes in the mRNA levels of the BM collagens coincide with the acquisition of invasive growth properties.

## Methods

### Subject population

The KAM cohort (Kolorektal cancer, Arv og Miljø) is based primarily on the screening group of the Norwegian Colorectal Cancer Prevention study (the NORCCAP study) in the county of Telemark, Norway [21]. Additionally, a series of colorectal cancer cases was recruited to the KAM cohort from routine clinical work at Telemark Hospital in Skien and Ullevål University Hospital in Oslo. In the NORCCAP study a total of 20,780 men and women, age distribution 50–64 years, drawn randomly from the population registries in Oslo (urban) and the county of Telemark (mixed urban and rural) were invited to have a flexible sigmoidoscopy (FS) screening examination with or without (1:1) an additional faecal occult blood test (FOBT). 777 (4%) individuals were excluded as previoualy described [21]. The KAM biobank currently consists of 234 colorectal cancer cases, 1044 cases with adenomas (229 high-risk, 762 low-risk and 53 hyperplastic polyps) and 400 controls. Controls were defined as individuals with normal findings at FS. In the present study we analyzed cases with carcinoma (n = 33) and adenoma (n = 29). For individuals with adenoma a sample of control tissue was collected 30 cm from the anus. For patients with carcinoma two samples of control tissue were taken from the surgical specimen, one sample in close proximity (normal adjacent), and one sample as far away from the tumour as possible (normal distant). Control samples from healthy controls were taken from individuals (n = 20) where no adenomas or carcinomas could be identified with FS. The histology was examined independently by two histopathologists. The specimens were put into liquid nitrogen as fast as possible after surgical removal. The colorectal cancer patients were classified according to Dukes system (A: Invasion but not through the bowel wall. B: Invasion but through the bowel wall but not involving lymph nodes. C: Involvement of lymph nodes. D: Widespread metastases.)

The KAM study is approved by the Regional Committee for Medical Research Ethics and the Norwegian Data Inspectorate, and informed consent has been obtained. The ID number for the NORCCAP study at Clinicaltrials.gov is NCT00119912 [22].

### RNA purification and cDNA synthesis

Total RNA was purified from tissue as recommended by the manufacturers using E.Z.N.A. Total RNA Kit II (VWR, Copenhagen, Denmark). The tissue had been stored in liquid N_2 _before RNA purification. RNA purification included a DNAse treatment. The cDNA synthesis was performed on approximately 200 ng RNA per 20 μl using High-Capacity cDNA Archive Kit (Applied Biosystems).

### Quantitative RT-PCR

Quantitative RT-PCR was performed using SYBR^® ^Green. All assays were performed using the ABI 7300 Sequence Detection System (Applied Biosystems).

Primers were obtained from TAG Copenhagen.*α1(IV) *F, 5'-CAG CCA GAC CAT TCA GAT CC-3';*α1(IV) *R, 5'-GGC GTA GGC TTC TTG AAC AT-3' [12]. *α4(IV) *F, 5'-AGA GAT TGC TCT GTT TGC CAC-3'; *α4(IV) *R, 5'-CGG TCC CCT CTC ATT CCT T-3' (Primerbank). *α6(IV) *F, 5'-CTC CTT GCC CTC ACT CAT AGC-3'; *α6(IV) *R, 5'-GTC TCC CTT AGG CCC TTT AGG-3' (Primerbank) *α1(VII) *F, 5'-CGG AAC TGA CCA TCC AGA AT-3'; – 3' *α1(VII) *R, 5'-AAT AGG GTG CTC ACG GTC AC-3' [23]. *β-actin *F, 5'-CTG GCA CCC AGC ACA ATG-3'; *β-actin *R, 5'-AGC GAG GCC AGG ATG GA-3' (Primer Express 3.0). The PCR reactions were performed using 900/50/900/300/900 nM F primer and 900/300/300/300/900 nM R primers for α1(IV), α4(IV), α6(IV), α1(VII) and β-actin respectively. Quantitative Power SYBR^® ^Green PCR master mix (Applied Biosystems) was used accordingly to the instructions from the manufacturer. To ensure that the PCR efficiency was equal for both the target gene and the reference gene, we made an efficiency plot with a serial dilution of cDNA. The validation showed that the assays are quantitative over a range of 256/128/16/64/256-fold dilution for α1(IV), α4(IV), α6(IV), α1(VII) provided that a threshold of 0.2 is used for all primer sets. The threshold is a fixed fluorescence signal level above the baseline, and the C_t _value of a sample is determined as the fractional cycle number where the sample's fluorescence signal exceeds the threshold. The collagens and β-actin were quantified in separate wells in duplicates. The standard deviation on repeated measurements of the same sample (the control) in separate experiments was 4%, 19%, 30% and 12% for α1(IV), α4(IV), α6(IV) and α1(VII) respectively, indicating the day-to-day variability of the assay. Negative controls (where the RNA is not converted into cDNA) and positive controls were included in all sets.

### Statistical analysis

MiniTab Statistical Software, Release 13.1 Xtra (Minitab Inc.), and GraphPad Prism 4 were used for the statistical calculations. The data were not adjusted for gender since the incidence ratio of colorectal cancer between the genders is 1:1 in Norway [24].

### Immunohistochemistry

The frozen, unfixed tissue sections were mounted on a precooled cryostat table by use of Tissue-Tek (Sakura Finetek). 7 μm thick sections were cut on a Leitz cryostat at -20°C and collected on glass slides. The sections were examined by immunofluorescence microscopy for the localization of type IV and type VII collagens using polyclonal rabbit anti type IV collagen (Abcam 6586) and monoclonal mouse anti-type VII collagen (Abcam 6312), respectively. All primary antibodies were diluted 1:100. Alexa Fluor goat anti-mouse IgG conjugate or Alexa Fluor goat anti-rabbit IgG conjugate (1:400 or 1:800) were used as secondary antibodies. Controls without primary antibody were run in parallel. The sections were finally mounted in antifade mounting medium, with or without DAPI (Vector Laboratories Inc.), and examined in a Leica DM 4000B microscope equipped with a Leica DC 300 FX camera.

## Results

**Figure 1:**
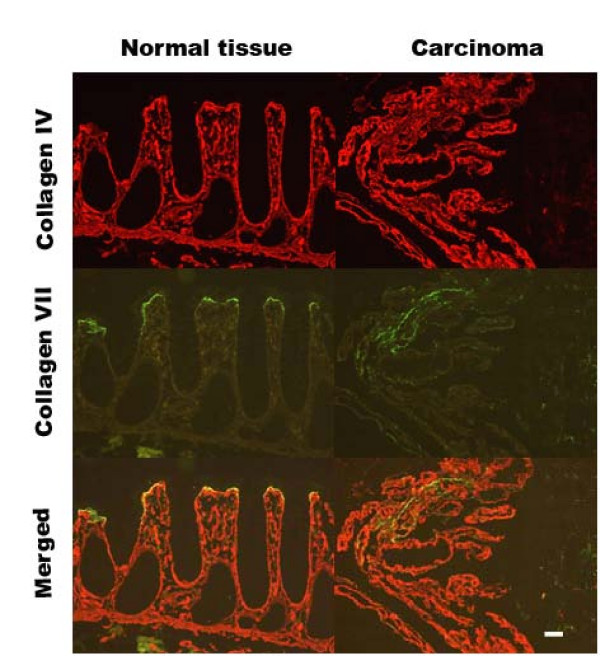
**Location of type IV collagen and type VII collagen in normal and colorectal cancer tissue from the same patient**. Frozen tissue sections were incubated on the glass slide with polyclonal rabbit anti-type IV collagen and monoclonal mouse anti-type VII collagen. Negative controls without primary antibody were run in parallel (not shown). The bar represents 50 μm.

The distribution of type IV and type VII collagen is shown in Fig 1. In normal tissue from controls and colorectal cancer patients type IV collagen was widely distributed in the extracellular matrix along the BM below the epithelial cells, in the perivascular stroma, and in the muscularis mucosa. In colorectal cancer tissue type IV collagen was detected in the same structures surrounding the distorted epithelial layer. In normal tissue type VII collagen could be detected at the BM of the surface epithelial cells, but could not be detected along the crypts or in other parts of the extracellular matrix. In the colorectal cancer tissue type VII collagen could be detected as a band below the epithelial cells in the distorted epithelium. None of the controls showed any staining.

The mRNA levels for *α1(IV), α4(IV), α6(IV)*, and *α1(VII) collagens *were measured in colon tissue samples from healthy control individuals (n = 20) and in healthy and affected tissue from individuals with adenomas (n = 29) and with colorectal cancer (n = 33) by real-time RT-PCR. Two histologically normal samples were analyzed from individuals with colorectal cancer, one "normal distant" taken as far away from the cancer tissue as allowed by the surgically removed tissue, and one "normal adjacent" taken immediately adjacent to the colorectal cancer tissue. We chose to normalize the mRNA levels of the *collagens *to the mRNA level of *β-actin*. The adenomas were characterized as mild dysplasia (n = 1), moderate dysplasia (n = 22) or non-determined (n = 6). The characteristics of the cases and healthy persons used in this study are shown in Table 1.

**Table 1:** Characteristics of cases and healthy persons used in this study

	Healthy	Cases	
		Adenomas	Carcinomas

	(n = 20)	(n = 29)	(n = 33)

Men	8	21	20

Women	12	8	13

Mean age +SD^1^	56.0 ± 4.6	56.9 ± 3.8	68.8 ± 11.4

^1^The patients with carcinoma are significantly older than the two other groups at 95% confidence level (Kruskal-Wallis test).

**Figure 2:**
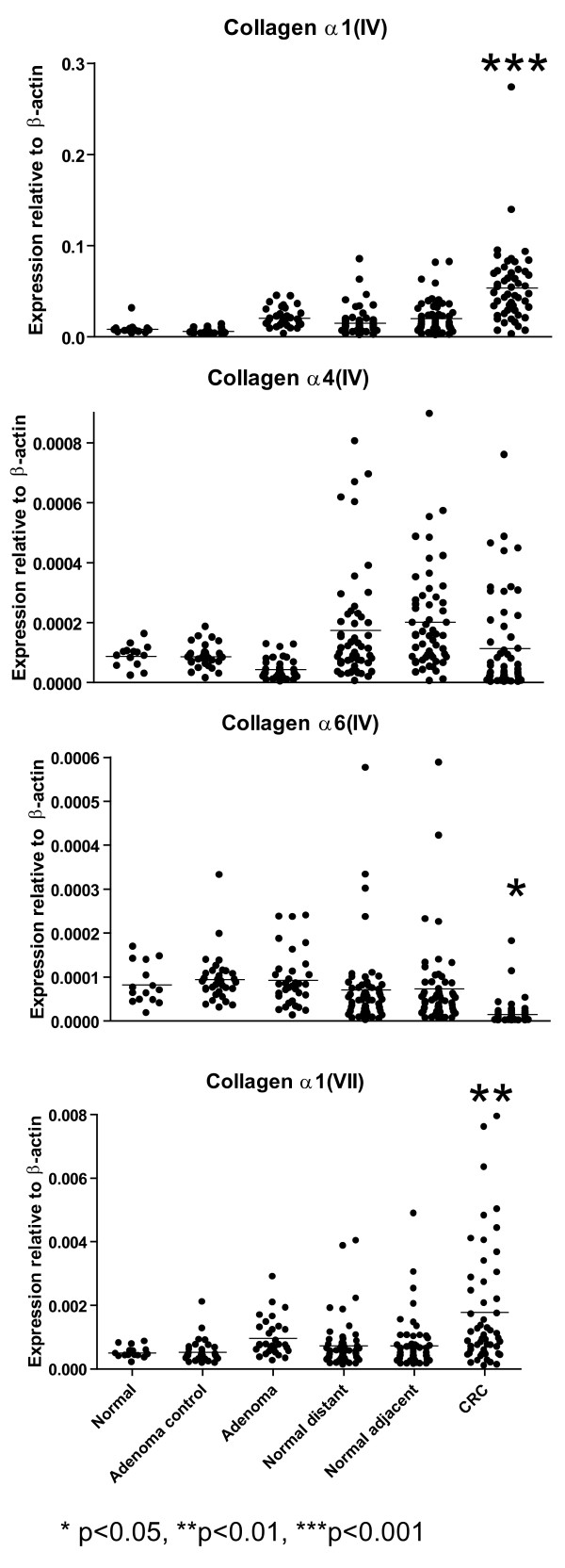
**The mRNA levels of mRNA for *α1(IV), α4(IV), α6(IV) *and *α1(VII) collagens *determined by real-time RT-PCR from healthy individuals (Normal), normal tissue from individuals with adenoma (Adenoma control), tissue from adenomas (Adenomas), normal tissue from colorectal cancer patients taken as far away from the carcinoma as possible from the surgically removed tissue (Normal distant), normal tissue from colorectal cancer patients taken near to the carcinoma (Normal adjacent) or colorectal cancer tissue (CRC)**. Each dot represents one individual. All mRNA levels were normalized to the level of *β-actin *mRNA.

mRNA levels for *α1(IV), α4(IV), α6(IV) *and *α1(VII) collagens *normalized to *β-actin *are shown in Fig. 2. The *α1(IV) *mRNA level was increased both in adenomas and colorectal cancer tissue. The *α1(IV) *mRNA level of colorectal cancer tissue was 6-fold higher than the corresponding tissue from healthy individuals (p < 0.001) (Table 2 and Fig. 2). When comparing affected tissue with normal tissue from the same individual, we found a statistically significant difference between individuals with adenomas (p < 0.001) and individuals with colorectal cancer when compared to both the normal distant and normal adjacent tissues (p < 0.001).

**Table 2:** *α1(IV)*, *α4(IV)*, *α6(IV) *and *α1(VII) *mRNA levels in normal and neoplastic colonic tissues.

	mRNA level in normal tissue* Mean (SD)	p^a^	mRNA level in neoplastic tissue Mean (SD)	p^a^	p^b^
***α1(IV)***					

Healthy individual	8.3 × 10^-3 ^(6.5 × 10^-3^)				

Patients with adenoma	6.3 × 10^-3 ^(2.6 × 10^-3^)	NS	20.6 × 10^-3 ^(10.4 × 10^-3^)	NS	< 0.001

Patients with carcinoma	15.0 × 10^-3 ^(14.6 × 10^-3^)**	NS	53.6 × 10^-3 ^(42.0 × 10^-3^)	< 0.001	< 0.001

Patients with carcinoma	20.2 × 10^-3 ^(17.8 × 10^-3^)***	NS	53.6 × 10^-3 ^(42.0 × 10^-3^)		< 0.001

***α4(IV)***					

Healthy individual	8.8 × 10^-5 ^(3.6 × 10^-5^)				

Patients with adenoma	8.6 × 10^-5 ^(3.7 × 10^-5^)	NS	4.4 × 10^-5 ^(3.5 × 10^-5^)	NS	< 0.001

Patients with carcinoma	17.5 × 10^-5^(18.7 × 10^-5^)**	NS	11.4 × 10^-5 ^(15.9 × 10^-5^)	NS	NS

Patients with carcinoma	20.1 × 10^-5 ^(16.9 × 10^-5^)***	NS	11.4 × 10^-5 ^(15.9 × 10^-5^)		< 0.01

***α6(IV)***					

Healthy individual	8.2 × 10^-5 ^(4.6 × 10^-5^)				

Patients with adenoma	9.4 × 10^-5 ^(5.6 × 10^-5^)	NS	9.3 × 10^-5 ^(6.3 × 10^-5^)	NS	NS

Patients with carcinoma	7.1 × 10^-5 ^(9.5 × 10^-5^)**	NS	1.5 × 10^-5 ^(2.8 × 10^-5^)	< 0.05	< 0.001

Patients with carcinoma	7.4 × 10^-5 ^(9.8 × 10^-5^)***	NS	1.5 × 10^-5 ^(2.8 × 10^-5^)		< 0.001

***α1(VII)***					

Healthy individual	5.1 × 10^-6 ^(1.8 × 10^-6^)				

Patients with adenoma	5.2 × 10^-6 ^(3.8 × 10^-6^)	NS	9.6 × 10^-6 ^(6.0 × 10^-6^)	NS	< 0.01

Patients with carcinoma	7.3 × 10^-6 ^(7.6 × 10^-6^)**	NS	17.8 × 10^-6 ^(1.9 × 10^-6^)	< 0.01	< 0.001

Patients with carcinoma	7.3 × 10^-6 ^(8.0 × 10^-6^)***	NS	17.8 × 10^-6 ^(1.9 × 10^-6^)		< 0.001

The mRNA levels were normalized to *β-actin *mRNA levels.

*Normal tissue: histologically normal colonic biopsies taken from healthy individuals or individuals with adenomas or carcinoma: ** = normal tissue taken from surgical specimens as distant from the carcinoma as possible; *** = normal tissue taken in close proximity to the carcinoma.

NS = not significant (P > 0.05)

a) p value for the comparison of the mRNA levels in normal tissue from healthy individuals with either normal or neoplastic tissue from patients using a one-way ANOVA and Tukey's Multiple Comparison Test.

b) p value for the comparison of the mRNA levels in normal and neoplastic tissue from the same patient using paired T-test.

mRNA level for *α4(IV) *displayed little change during carcinogenesis (Fig. 2). We found no statistically significant differences when comparing the level of *α4(IV) *mRNA in the various groups with corresponding tissue from healthy individuals. The mRNA level for *α4(IV) *was slightly but statistically significantly lower in adenomas when normal and affected tissue from the same individual was compared (p < 0.001). A similar decrease was observed when normal adjacent and colorectal cancer tissue were compared (p < 0.01). However, no statistically significant difference was seen when comparing the normal distant samples with colorectal cancer tissue.

mRNA level for *α6(IV) *was decreased in colorectal cancer tissue (Fig. 2.). Compared with the level of *α6(IV) *mRNA in tissue from healthy individuals there was a statistically significant 5-fold decrease in colorectal cancer tissue (p < 0.05). When comparing normal and affected tissue from the same individual statistically significant differences were seen in colorectal cancer patients both when compared to normal distant and normal adjacent tissue (p < 0.001). However, no differences in mRNA levels were detected when comparing normal and affected tissue from individuals with adenomas.

mRNA level for *Type VII collagen *was increased in both adenomas and colorectal cancer tissue (Fig. 2.). The level of mRNA for *α1(VII) collagen *was increased 3.5-fold in colorectal cancer tissue compared to normal tissue in healthy individuals (p < 0.01). mRNA for *α1(VII) collagen *was statistically significantly increased in individuals with adenomas (p < 0.01) and carcinomas (p < 0.001) when comparing affected and normal tissue from the same individual.

The colorectal cancer patients were staged as Dukes grade A (n = 6), grade B (n = 25) and grade C (n = 16). No Dukes grade D patients were found in this set of samples. No tendency and no statistically significant differences (one-way ANOVA and Tukey's post test for paired comparison) were seen when comparing the *α1(IV), α4(IV), α6(IV)*, and *α1(VII) *mRNA levels in the different Dukes groups.

## Discussion

In the present study we have determined the mRNA levels of *type IV collagen (chains α1(IV), α4(IV), α6(IV)) *and *type VII collagen (chain α1(VII)) *during colorectal cancer carcinogenesis.

Former studies on colorectal normal and neoplastic tissues have been performed on a relatively small number of patients often not including controls from healthy individuals. Furthermore, former studies have primarily used immunohistochemistry, a technique that can demonstrate the presence of the proteins but only in a semi-quantitative manner. In the present study we have included control samples from healthy individuals in addition to normal tissue from individuals with adenomas and carcinomas. In addition, we have used quantitative real time RT-PCR to indicate the levels of mRNAs coding for collagen chains. To the best of our knowledge this is the first quantitative assessment of changes in collagen mRNA levels during carcinogenesis.

Our results show, that the normal and neoplastic colon mucosa express *α1/α4/α6(IV) collagens*. This confirms earlier results obtained by immunohistochemistry staining using chain-specific monoclonal antibodies [7]. When examining the level of mRNA for *α1(IV) *we found an elevated level already in adenomas that is maintained in the colorectal cancer tissue compared to both corresponding tissue from healthy individuals and histologically normal tissue from the colorectal cancer patients. The change in *α1(IV) *mRNA levels detected in this study therefore do not coincide with acquisition of invasive growth properties. Since *α1(IV) *is widely distributed in the tissue, and not only in the subepithelial BM, its shown up-regulation in the neoplastic tissue might be connected to an increased amount of *α1(IV) *in stromal BMs as well. Immunohistological studies have previously suggested that *α1(IV) *is down-regulated in colorectal cancer in relation to tumour differentation [7]. However, when comparing tumours with different Dukes stages we did not show significant differences.

We found a diminished mRNA level for *α4(IV) *in adenomas and cancer tissue as compared to normal control tissue in the same patient. This result is in agreement with a previous study where α4(IV) was undetectable by immunohistochemistry in colon tumour biopsies, but was detectable in normal tissue from the same individual [7]. It has also been described that α4(IV) is partly lost in bronchioalveolar carcinoma of the lung but present in histologically normal adjacent tissue [15]. α4(IV) collagen is localized exclusively in the BM below the colonic epithelial surface and might thus be of interest regarding invasiveness of malignant neoplasms. However, we found that the change of *α4(IV) *mRNA level is rather small and do not coincide with acquisition of invasive growth properties.

By quantitative RT-PCR we found that the mRNA level for *α6(IV) *was significantly down-regulated in colorectal cancer tissue compared to both healthy individuals and normal control tissue in the patients with carcinoma. There was no significant reduction in adenoma tissue. The loss of *α6(IV) *is in agreement with suggestions from earlier immunohistochemical studies in colorectal carcinoma [7,11]. In addition, loss of or the diminished presence of α5–6(IV) collagens have been demonstrated by immunohistochemistry in prostatic carcinoma [10], in basal cell carcinoma [25], in lung adenocarcinoma [15], and in gastric intramucosal carcinoma [8]. In contrast to α1(IV) which is widely distributed, α6(IV) is localized exclusively to the subepithelial BM along the luminal surface and the crypts in the colon [7] and is therefore of special interest regarding the question of invasive growth. In this study we find that the down-regulation of the level of mRNA for *α6(IV) *is a late event in carcinogenesis that coincides with acquisition of invasive growth properties. We therefore suggest that down-regulation of *α6(IV) *might be an important factor for acquisition of invasive growth properties of the tumour.

Our immunohistochemical studies show that type VII collagen is present in normal and neoplastic colonic tissue. Our quantitative real-time RT-PCR results show for the first time that there is a significant up-regulation of the level of mRNA for *α1(VII) collagen *in colorectal cancer tissue compared to both corresponding tissue from healthy individuals and normal tissue from patients with carcinoma. We found that individuals with adenomas have a significant increase in the level of *α1(VII) *mRNA in the adenoma tissue compared to normal tissue from the same individual. There are no significant differences between tissue from healthy individuals and normal tissue taken from individuals with adenomas or carcinomas. The up-regulation of *α1(VII) *mRNA is thus already seen in individuals with adenomas and maintained in carcinomas. The pattern of mRNA levels is very similar to the pattern seen for *α1(IV)*. However, as seen by histochemistry type VII collagen is localized solely beneath the surface epithelial cells and therefore – in contrast to α1(IV) specifically reflects alterations along the epithelial BM. Interestingly, the same localization is described for α3/α4(IV) by the use of chain specific antibodies [7]. It might be speculated whether up-regulation of *α1(VII) *in concert with down-regulation of *α4(IV) *results in alterations of importance for invasiveness.

Type VII collagen is also known as the epidermolysis bullosa acquisita antigen since patients with this disease have IgG auto-antibodies against type VII collagen. A few other studies have shown the presence of type VII collagen in normal colon [19,20]. Visser et al., (1993) demonstrated type VII collagen immunoreactivity in the BM zone in adenomas and adenocarcinomas but not in normal colon epithelium from 4 control patients and suggested that type VII collagen is involved in the process of early invasion [17]. We find that the up-regulation of *α1(VII) *mRNA occurs as an early event prior to the acquisition of invasive growth. We therefore suggest that up-regulation of *α1(VII) *mRNA might be a necessary but not sufficient event in the process of carcinogenesis.

The fact that there are no significant differences between tissue from healthy individuals and the normal tissue taken from the individual with adenomas or carcinomas suggests that it is not a primary defect in the mRNA levels of the single type IV collagen chains or type VII collagen that leads to development of cancer.

## Conclusion

In conclusion, we found that the mRNA levels of *α6(IV*) was down-regulated as a late event that coincides with the acquisition of invasive growth properties. *α1(IV) *and *α1(VII) *mRNAs were increased already in adenoma tissue and maintained at a high level in colorectal cancer tissue. A change that may be necessary but not sufficient for acquisition of invasive growth properties. Future studies are required to clarify whether changes in collagen composition are a cause or a consequence of malignant progression.

## Competing interests

The authors declare that they have no competing interests.

## Authors' contributions

HS conceived the idea of the study and designed the collagen primers. HS and DA performed the immunohistochemistry. EHK designed and administered the KAM study and collected the samples. IMBL evaluated the tissue sections. KMT contributed with scientific advice to the KAM study. LV and DA carried out and evaluated the RT-PCR. LV performed the statistical evaluation. All authors helped interpret the results, writing the manuscript and approved the final version.

## Pre-publication history

The pre-publication history for this paper can be accessed here:

http://www.biomedcentral.com/1471-2407/9/136/prepub

## References

[B1] Kalluri R Basement membranes: structure, assembly and role in tumour angiogenesis Nat Rev Cancer 2003 3 422 433 10.1038/nrc1094 12778132

[B2] Timpl R Structure and biological activity of basement membrane proteins Eur J Biochem 1989 180 487 502 10.1111/j.1432-1033.1989.tb14673.x 2653817

[B3] Mariyama M Leinonen A Mochizuki T Tryggvason K Reeders ST Complete primary structure of the human alpha 3(IV) collagen chain. Coexpression of the alpha 3(IV) and alpha 4(IV) collagen chains in human tissues J Biol Chem 1994 269 23013 23017 8083201

[B4] Ninomiya Y Kagawa M Iyama K Naito I Kishiro Y Seyer JM Sugimoto M Oohashi T Sado Y Differential expression of two basement membrane collagen genes, COL4A6 and COL4A5, demonstrated by immunofluorescence staining using peptide-specific monoclonal antibodies J Cell Biol 1995 130 1219 1229 7657706 10.1083/jcb.130.5.1219 PMC2120565

[B5] Sado Y Kagawa M Naito I Ueki Y Seki T Momota R Oohashi T Ninomiya Y Organization and expression of basement membrane collagen IV genes and their roles in human disorders J Biochem 1998 123 767 776 9562604 10.1093/oxfordjournals.jbchem.a022003

[B6] Simoneau A Herring-Gillam FE Vachon PH Perreault N Basora N Bouatrouss Y Pageot LP Zhou J Beaulieu JF Identification, distribution, and tissular origin of the alpha5(IV) and alpha6(IV) collagen chains in the developing human intestine Dev Dyn 1998 212 437 447 10.1002/(SICI)1097-0177(199807)212:3<437::AID-AJA11>3.0.CO;2-Y 9671947

[B7] Oka Y Naito I Manabe K Sado Y Matsushima H Ninomiya Y Mizuno M Tsuji T Distribution of collagen type IV alpha1-6 chains in human normal colorectum and colorectal cancer demonstrated by immunofluorescence staining using chain-specific epitope-defined monoclonal antibodies J Gastroenterol Hepatol 2002 17 980 986 10.1046/j.1440-1746.2002.02789.x 12167119

[B8] Baba Y Iyama K Ikeda K Ishikawa S Hayashi N Miyanari N Honda Y Sado Y Ninomiya Y Baba H Differential expression of basement membrane type IV collagen alpha chains in gastric intramucosal neoplastic lesions J Gastroenterol 2007 42 874 880 10.1007/s00535-007-2112-2 18008031

[B9] Baba Y Iyama K Ikeda K Ishikawa S Hayashi N Miyanari N Sado Y Ninomiya Y Baba H The expression of type IV collagen alpha6 chain is related to the prognosis in patients with esophageal squamous cell carcinoma Ann Surg Oncol 2008 15 555 565 10.1245/s10434-007-9592-4 17955302

[B10] Dehan P Waltregny D Beschin A Noel A Castronovo V Tryggvason K De Leval J Foidart JM Loss of type IV collagen alpha 5 and alpha 6 chains in human invasive prostate carcinomas Am J Pathol 1997 151 1097 1104 9327743 PMC1858039

[B11] Hiki Y Iyama K Tsuruta J Egami H Kamio T Suko S Naito I Sado Y Ninomiya Y Ogawa M Differential distribution of basement membrane type IV collagen alpha1(IV), alpha2(IV), alpha5(IV) and alpha6(IV) chains in colorectal epithelial tumors Pathol Int 2002 52 224 233 10.1046/j.1440-1827.2002.01341.x 11972866

[B12] Ikeda K Iyama K Ishikawa N Egami H Nakao M Sado Y Ninomiya Y Baba H Loss of expression of type IV collagen alpha5 and alpha6 chains in colorectal cancer associated with the hypermethylation of their promoter region Am J Pathol 2006 168 856 865 16507901 10.2353/ajpath.2006.050384 PMC1606532

[B13] Martinella-Catusse C Polette M Noel A Gilles C Dehan P Munaut C Colige A Volders L Monboisse JC Foidart JM Birembaut P Down-Regulation of MT1-MMP expression by the alpha3 chain of type IV collagen inhibits bronchial tumor cell line invasion Lab Invest 2001 81 167 175 11232638 10.1038/labinvest.3780224 PMC2966475

[B14] Misumi S Iyama K Honda Y Kitano T Sado Y Ninomiya Y Shinohara M Differential expression of basement membrane type-IV collagen alpha1, alpha2, alpha5 and alpha6 chains among the histological subtypes of adenoid cystic carcinoma Virchows Arch 2004 445 54 62 15138813 10.1007/s00428-004-1015-3

[B15] Nakano KY Iyama KI Mori T Yoshioka M Hiraoka T Sado Y Ninomiya Y Loss of alveolar basement membrane type IV collagen alpha3, alpha4, and alpha5 chains in bronchioloalveolar carcinoma of the lung J Pathol 2001 194 420 427 10.1002/path.928 11523049

[B16] Tanjore H Kalluri R The role of type IV collagen and basement membranes in cancer progression and metastasis Am J Pathol 2006 168 715 717 16507886 10.2353/ajpath.2006.051321 PMC1606530

[B17] Visser R Arends JW Leigh IM Bosman FT Patterns and composition of basement membranes in colon adenomas and adenocarcinomas J Pathol 1993 170 285 290 10.1002/path.1711700311 8133402

[B18] Christiano AM Greenspan DS Lee S Uitto J Cloning of human type VII collagen. Complete primary sequence of the alpha 1(VII) chain and identification of intragenic polymorphisms J Biol Chem 1994 269 20256 20262 8051117

[B19] Chen M O'Toole EA Sanghavi J Mahmud N Kelleher D Weir D Fairley JA Woodley DT The epidermolysis bullosa acquisita antigen (type VII collagen) is present in human colon and patients with crohn's disease have autoantibodies to type VII collagen J Invest Dermatol 2002 118 1059 1064 10.1046/j.1523-1747.2002.01772.x 12060403

[B20] Lohi J Leivo I Tani T Kiviluoto T Kivilaakso E Burgeson RE Virtanen I Laminins, tenascin and type VII collagen in colorectal mucosa Histochem J 1996 28 431 440 10.1007/BF02331434 8863048

[B21] Gondal G Grotmol T Hofstad B Bretthauer M Eide TJ Hoff G The Norwegian Colorectal Cancer Prevention (NORCCAP) screening study: baseline findings and implementations for clinical work-up in age groups 50–64 years Scand J Gastroenterol 2003 38 635 642 10.1080/00365520310003002 12825872

[B22] Clinical Trials 2007 http://clinicaltrials gov

[B23] Wang TW Sun JS Huang YC Wu HC Chen LT Lin FH Skin basement membrane and extracellular matrix proteins characterization and quantification by real time RT-PCR Biomaterials 2006 27 5059 5068 10.1016/j.biomaterials.2006.05.004 16781770

[B24] Norway TCR Cancer in Norway 2004. Oslo, Infoprint 2006 http://www.kreftregisteret.no/en/

[B25] Tanaka K Iyama K Kitaoka M Ninomiya Y Oohashi T Sado Y Ono T Differential expression of alpha 1(IV), alpha 2(IV), alpha 5(IV) and alpha 6(IV) collagen chains in the basement membrane of basal cell carcinoma Histochem J 1997 29 563 570 10.1023/A:1026428010104 9279559

